# A fatal course of COVID-19 during the Omicron surge: can you estimate your patients’ SARS-CoV-2 immune status?

**DOI:** 10.1007/s15010-023-02120-w

**Published:** 2023-11-06

**Authors:** Martin Mersch, Stefan Schlabe, Sven Breitschwerdt, Judith Laufenberg, Dorian Emmert, Souhaib Aldabbagh, Christoph Boesecke, Malte Benedikt Monin

**Affiliations:** 1 Department of Internal Medicine I, University Hospital Bonn, Venusberg-Campus 1, 53127 Bonn, Germany; 2 German Centre for Infection Research (DZIF), Partner-Site Cologne-Bonn, Bonn, Germany; 3 Institute of Virology, Bonn University Hospital, Bonn, Germany

**Keywords:** COVID-19, Omicron variant of concern, Severe course, Immune status

## Abstract

We present a case of an ultimately fatal course of COVID-19 (coronavirus disease-19) in an 81-year-old female patient during the Omicron surge. The patient did not represent the typical patient at risk for severe COVID-19 with significant causes of immunodeficiency. However, she had been skeptical about the vaccination for severe acute respiratory syndrome virus-2 (SARS-CoV-2) and had refused it. Moreover, there had been no previous COVID-19 episodes. Our case report illustrates that with regard to SARS-CoV-2, immunologically naive patients are still at risk for severe and/or even fatal courses of COVID-19. We call to implement both, recommendations for SARS-CoV-2 vaccinations as well as for antiviral treatment.

## Introduction

The COVID-19 (coronavirus disease-19) pandemic is less present in the public debate, and pandemic-related restrictions have also been reduced steadily—particularly during the Omicron surge. This variant of the severe acute respiratory syndrome virus-2 (SARS-CoV-2) is thought to be less affecting the patients overall. However, rates of up to 7.8% of severe courses have been described especially in patients with hematological malignancies [1].

## Case report

**Fig. 1:**
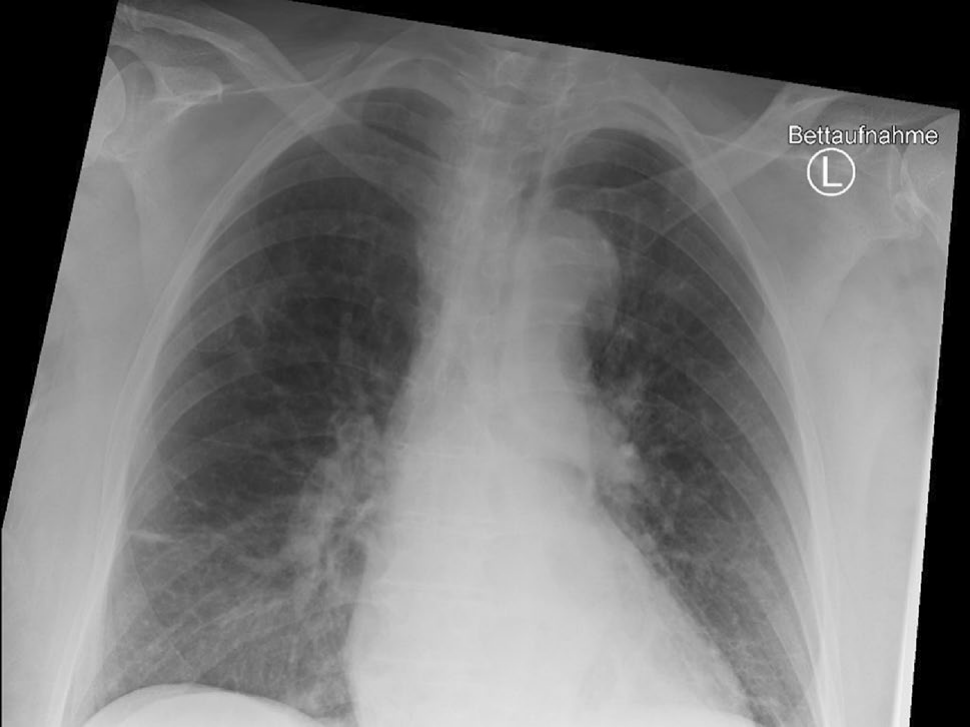
Chest X-ray on admission. Chest X-ray on admission showed basally accentuated bipulmonary infiltrates with signs of incipient pulmonary venous congestion

Here, we present a fatal case of an 81-year-old female patient. Before admission to the hospital, the patient lived alone, being supported in daily routine by family members. The patients’ past medical history included a mild dementia due to microangiopathic changes, an essential tremor, and a left coxarthrosis. A hip replacement was performed in March 2023. Subsequently, the patient was transferred to a rehabilitation facility. There, she developed cough, fever up to 39.2 °C and dyspnea on exertion, and was tested positive for SARS-CoV-2 with a minimum cycle threshold of 15,81. In the further course, it became necessary to start supplemental oxygen via a nasal cannula. Finally, the patient was relocated to our university hospital. On admission, we saw a cardio-pulmonary stable patient. With the administration of 4 L of oxygen via a nasal cannula, a peripheral oxygen saturation of 95% could be reached. Laboratory findings showed elevations of C-reactive protein (CRP) to 115 mg/l (reference 0–3 mg/l) and of lactate dehydrogenase (LDH) to 389 U/l (reference < 250 U/l) as well as a leukocytopenia of 2.81 G/l (reference 3.6–10.5 G/l). The changes were considered typical for COVID-19. Chest X-ray showed basally accentuated bipulmonary infiltrates with signs of incipient pulmonary venous congestion (Fig. 1). The SARS-CoV-2 sublineage XBB.1.5 was determined by polymerase chain reaction and subsequent next generation sequencing.

Medical history revealed that the patient had been skeptical about the vaccination for SARS-CoV-2 and had refused it. Moreover, there had been no previous COVID-19 episodes. The possibility of an antiviral therapy with remdesivir was endorsed, but the patient denied it. As a consequence, a therapeutic limitation was set in the patient’s interest (no resuscitation, no transfer to an intensive care unit, and no invasive ventilation).

**Fig. 2:**
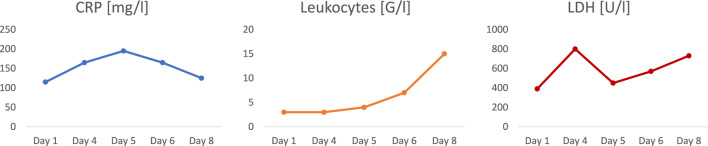
Laboratory findings over time. Laboratory findings indicating a severe course of COVID-19 with elevation of CRP and LDH as well as an inital leukocytopenia followed by a leucocytosis. Abbreviations: *CRP* c-reactive protein, *LDH* lactate dehydrogenase

On the fourth day after admission, an increase in oxygen demand, being accompanied with a rise CRP-level and markedly elevated LDH (Fig. 2), was noted. Echocardiography showed no evidence of right heart strain, so pulmonary artery embolism was not assumed. Sonography was used to exclude pleural effusions. There were no clinical and/or laboratory signs of sepsis. An immunomodulatory therapy with dexamethasone was initiated. On the following day, the patient was additionally started on piperacillin/tazobactam (4.0 g/0.5 g, intravenous application every 8 h), to treat a potential bacterial superinfection. Still, no septic course could be detected. At that moment, she already required a continuous oxygen supplementation via a non-rebreather mask to achieve a peripheral oxygen saturation of > 90%. A re-evaluation of the therapy goal was performed, in which the therapy limitation was reconfirmed. On day 11 after admission, she passed due to a global respiratory failure, being under palliative care at that time.

## Discussion

Our case report illustrates that with regard to SARS-CoV-2, immunologically naive patients are still at risk for severe and/or even fatal courses of COVID-19. Of note, our patient did not represent the typical patient at risk for severe COVID-19 with hematological malignancies or other causes of immunodeficiency. We could recently show that age over 65 years is another outstanding predictor for severe courses of COVID-19 [2], which this case underlines. Our case makes an important contribution to the published case reports and smaller case series, especially as infection rates are expected to rise in autumn/winter and with reduced hygienic measures increasing the danger for vulnerable patients.

In Germany, 77.9% of the population is vaccinated according to the recommendations of the German expert panel (*Ständige Impfkommission*: STIKO) to date [3]. Therefore, despite all efforts, almost a quarter of the population has an incomplete and, thus, unreliable vaccination protection. Of the patients treated for COVID-19 in an intensive care unit, 12.9% (*n* = 82/634) were not vaccinated in May 2023 [4]. Furthermore, it has been shown that after a full course of vaccination, the titers of SARS-COV-2 anti-spike IgG decrease over time which results in an insufficient protection status [5]. Due to subclinical courses of COVID-19 as well as the overall decrease in testing for infections with SARS-CoV-2, no really reliable numbers of previously uninfected persons can be given. Taking these information into consideration, it is important to assess at least the approximate SARS-CoV-2 immune status in every single patient after vaccination for further decisions. However, serological testing should only be discussed in patients at high risk [6].

The fatal course of COVID-19 in our patient demonstrates that patients with insufficient vaccine protection should be encouraged to close the individual vaccination gap. At the moment, the STIKO recommends basic immunization and booster vaccination, which applies to everyone older than 18 years of age regardless of any underlying diseases. Thus, everyone should undergo three antigen presentations, at least two of which should consist of vaccinations. At an interval of 12 months from the last exposure to the antigen, people at risk should be re-vaccinated [7]. It is important to implement the current vaccination recommendations of the STIKO so that patients with SARS-CoV-2 infection do not suffer avoidable severe complications.

Despite vaccination, patients can still develop severe courses of COVID-19. Those patients should receive early antiviral therapy as recommended by current guidelines [8, 9]. If the opportunity for early antiviral therapy has been missed, remdesivir can be given and/or immunomodulatory therapy considered at an advanced stage [2, 8–10].

In summary, the development of vaccines and antiviral agents against SARS-CoV-2 is a success story. Implementation should now be promoted.

## Data Availability

Data are available upon request from corresponding author.
